# Using observational study data as an external control group for a clinical trial: an empirical comparison of methods to account for longitudinal missing data

**DOI:** 10.1186/s12874-022-01639-0

**Published:** 2022-05-28

**Authors:** Vibeke Norvang, Espen A. Haavardsholm, Sara K. Tedeschi, Houchen Lyu, Joseph Sexton, Maria D. Mjaavatten, Tore K. Kvien, Daniel H. Solomon, Kazuki Yoshida

**Affiliations:** 1 Division of Rheumatology and Research, Diakonhjemmet Hospital, Box 23 Vinderen, 0319 Oslo, Norway; 2 Division of Rheumatology, Inflammation, and Immunity, Department of Medicine, Brigham and Women’s Hospital/Harvard Medical School, Boston, USA; 3 Faculty of Medicine, University of Oslo, Oslo, Norway; 4 Harvard Medical School, Boston, USA; 5 Department of Orthopedics, Chinese PLA General Hospital, Beijing, China

**Keywords:** External control group, Missing data, Multiple imputation, Inverse probability weighting

## Abstract

**Background:**

Observational data are increasingly being used to conduct external comparisons to clinical trials. In this study, we empirically examined whether different methodological approaches to longitudinal missing data affected study conclusions in this setting.

**Methods:**

We used data from one clinical trial and one prospective observational study, both Norwegian multicenter studies including patients with recently diagnosed rheumatoid arthritis and implementing similar treatment strategies, but with different stringency. A binary disease remission status was defined at 6, 12, and 24 months in both studies. After identifying patterns of longitudinal missing outcome data, we evaluated the following five approaches to handle missingness: analyses of patients with complete follow-up data, multiple imputation (MI), inverse probability of censoring weighting (IPCW), and two combinations of MI and IPCW.

**Results:**

We found a complex non-monotone missing data pattern in the observational study (*N* = 328), while missing data in the trial (*N* = 188) was monotone due to drop-out. In the observational study, only 39.0% of patients had complete outcome data, compared to 89.9% in the trial. All approaches to missing data indicated favorable outcomes of the treatment strategy in the trial and resulted in similar study conclusions. Variations in results across approaches were mainly due to variations in estimated outcomes for the observational data.

**Conclusions:**

Five different approaches to handle longitudinal missing data resulted in similar conclusions in our example. However, the extent and complexity of missing observational data affected estimated comparative outcomes across approaches, highlighting the need for careful consideration of methods to account for missingness in this setting. Based on this empirical examination, we recommend using a prespecified advanced missing data approach to account for longitudinal missing data, and to conduct alternative approaches in sensitivity analyses.

**Supplementary Information:**

The online version contains supplementary material available at 10.1186/s12874-022-01639-0.

## Introduction

Data from observational studies and registries are increasingly being used to complement randomized controlled trials (RCTs) in clinical effectiveness research [1–3]. One recognized approach is to use observational data as an external control group to compare with clinical trial data [2–6]. The external controls may be historical or contemporaneous and may represent the natural course of disease or current standard of care [4, 5]. However, integrating trial data and observational data in one comparative effectiveness study poses methodological challenges due to the heterogeneity of data sources, not only related to the lack of randomization, but also due to differences in follow-up data [4–9].

The follow-up strategy in a clinical trial will typically include more frequent and rigorous monitoring compared with an observational study carried out in clinical practice [1, 4, 8]. This can result in differential patterns of longitudinal missing data. Addressing these differences is crucial to limit potential selection bias when using observational data sources to form external control groups for clinical trials [4, 5, 9, 10]. However, guidance in how to assess and address longitudinal missing data in this setting is scarce.

The present methodological investigation was motivated by challenges arising in a previous study in which we used data from a prospective observational study as an external contemporaneous control group to be compared with a clinical trial [11]. The objectives of this paper are to 1) describe differences in missing data patterns during follow-up in a clinical trial versus an observational study and 2) empirically examine the impact of different missing data methods on study conclusions when using observational study data to form an external control group for a clinical trial.

## Methods

In the following, we explain the clinical setting and data sources that motivated our methodological investigation, the description of longitudinal missing patterns, and the empirical comparison study. All statistical methods were carried out using the statistical software STATA, version 16.0.

### Clinical setting and data sources

Data for the present methodological investigation were provided by the ARCTIC trial [12] and the NOR-VEAC prospective observational study [11, 13]. Both enrolled patients with recently diagnosed rheumatoid arthritis (RA) and implemented treat-to-target strategies of different stringency as patients started disease-modifying anti-rheumatic drug (DMARD) therapy.

“Treat-to-target” in RA care involves defining a disease activity treatment target when initiating a new therapy, frequent monitoring and adjusting therapy if the target is not achieved [14]. Disease activity is typically measured by a composite score calculated from clinically relevant measurements, such as the number of swollen and tender joints, the patient’s global assessment of disease, and an inflammatory biomarker [15]. The preferred treatment target in RA is remission, i.e., a clinical state of no or little remaining disease activity [14, 16]. Treat-to-target is currently the recommended approach in the care for patients with RA [16, 17], however, the stringency of the strategy (i.e., how often to conduct follow-up) and how aggressive the disease activity target should be is debated.

The ARCTIC trial [12] included 230 RA-patients at eleven rheumatology centers across Norway during the period 2010–2013. Patients were scheduled to meet for thirteen visits during two years of follow-up from the time point of initiating DMARD therapy. All patients were treated according to the same pre-specified treatment escalation protocol. The ARCTIC trial was designed to examine the benefit of structured ultrasound assessments compared with conventional follow-up under the principles of the same treat-to-target strategy. As the two original strategies of the trial showed similar outcomes in the main study [12], we pooled the groups for the purpose of the present study, giving “single-arm” trial data. The ARCTIC strategy represents a stringent treat-to-target strategy for RA.

The NOR-VEAC prospective observational study [11, 13] included 429 RA-patients at five rheumatology centers in Norway in the period 2010–2016. Patients were prospectively followed from the time of initiating DMARD therapy. Monthly visits were scheduled until the treatment target had been achieved, followed by visits every 3–6 months. The study protocol in the NOR-VEAC study did not specify a treatment escalation algorithm; however, participating study centers committed to follow current international treat-to-target recommendations [14, 18]. The NOR-VEAC strategy represents a less stringent and more pragmatic treat-to-target strategy.

Patients were included for the present study according to a common set of eligibility criteria (supplementary file S1).

### Baseline balancing and longitudinal missing data in target trial emulation

Defining and identifying missing data during follow-up requires an understanding of what “ideal” follow-up data might look like. This is not straightforward when using observational data sources as external controls for a clinical trial. We approached this issue using the target trial emulation framework. We conceptualized a hypothetical target trial [19] that compares stringent and pragmatic treat-to-target strategies for RA (supplementary file S2). To emulate baseline randomization to either the ARCTIC trial (representing the stringent treat-to-target strategy) or the NOR-VEAC observational study (representing the pragmatic treat-to-target strategy), we used inverse probability of treatment weighting using the propensity scores calculated from baseline covariates [20, 21]. The propensity scores were calculated by regressing assignment to either the ARCTIC strategy or the NOR-VEAC strategy on the following baseline covariates considered to be potential confounders in the estimation of the main outcome: age, gender, months since first swollen joint, higher education (completed college or university degree), rheumatoid factor positivity, anti-cyclic citrullinated peptide positivity, erythrocyte sedimentation rate, C-reactive protein, swollen and tender joint count in 28 joints, patient global assessment of disease, physician global assessment of disease, number of comorbidities (≥1 versus none), smoking status (never/previous vs current), fatigue (VAS 0–100) and the EQ-5D-index score. Further details on the IPTW model and the corresponding STATA code are available in supplementary file S3. Time zero, or baseline, was set to the time of initiating DMARD therapy, which occurred at or shortly after inclusion for both studies.

The ideal data from the target trial would have all patients’ outcome data at the 6, 12 and 24-months follow-up visits. However, missing outcome data occurred in both “arms” of the empirical example. To enable the use of appropriate missing data methods to account for missingness, we separately identified and assessed three types of missingness in the trial data arm and in the observational data arm (supplementary file S4). The first type of missingness is “drop-out,”, which arises from loss to follow-up. This type of missingness is common in longitudinal studies and, if occurring alone, it results in a monotone missing data pattern, i.e., patients are followed with complete data until they drop out of the study [22, 23]. A second type of missingness is “intermittent missing visits”, which can arise when patients do not meet for one or more visits, but reenter the study on a later time point [22, 23]. This type of missingness may also arise from the misalignment of visits occurring in the empirical data and the study visits in the target trial. Although both the trial and the observational study had scheduled visits at the selected time points (6,12 and 24 months after baseline), some patients in the observational study may have completed the scheduled visit on a later time point. A third type of missingness is “missing outcome data at visit”. In this type of missingness, patients do have visits corresponding to the study visits that the target trial dictates. However, one or more of the components of the composite outcome score are missing. Intermittent missing visits and missing outcome data at visits results in a non-monotone missing data pattern [22, 23]. In longitudinal studies, monotone and non-monotone data patterns are often observed simultaneously, especially in studies with an observational design [23].

### Missing data approaches

We empirically evaluated five different approaches to handle these three types of missing data, as displayed in Table 1. In this section we outline the approaches and evaluation metrics.

**Table 1:** Evaluated approaches to missing outcome data during follow-up when using data from an observational study as an external control arm to a clinical trial

Approach	Missing outcome data at visit^**f**^	Intermittent missing visits^**g**^	Drop-outs^**h**^
Complete follow-up case analyses^a^	Exclusion	Exclusion	Exclusion
Strict censoring + IPCW^b^	Censoring (set as drop-out)	Censoring (set as drop-out)	IPCW
MI + censoring + IPCW^c^	MI	Censoring (set as drop-out)	IPCW
MI + IPCW^d^	MI	MI	IPCW
MI for all^e^	MI	MI	MI

*IPCW* Inverse probability of censoring weighting, *MI* Multiple imputation

^a^Assumptions: Patients with complete follow-up data are exchangeable with patients with missing data

^b^ Assumptions: The IPCW model is correctly specified when modeling the missing mechanism given previous observations in individuals with missing data due to drop-out (naturally occurring or created due to artificial censoring)

^c^ Assumptions: Both the MI model and the IPCW model are correctly specified. MI models missing outcome variables at visits given available information in the dataset. IPCW models the missing mechanism given the observed past for missing data due to drop-out (naturally occurring or created due to artificial censoring)

^d^ Assumptions: Both the MI model and the IPCW model are correctly specified. MI models missing outcome variables at visits and intermittent missing visits given information in the dataset. IPCW models the missing mechanism given the observed past for missing data due to naturally occurring drop-out

^e^Assumptions: The MI model is correctly specified when modeling all missing outcome data given information in the dataset. Separate models were specified for each cohort and the imputed datasets were thereafter combined

^f^A visit is recorded at the required time point, but the disease activity measure is missing

^g^A visit is missing for the 6-month and/or 12-month follow-up, but there is a later visit within the timeframe of the study

^h^A follow-up visit and all subsequent visits are missing within the timeframe of the study

#### Complete follow-up case analysis

In this approach, we performed analyses in a subset of patients with complete follow-up data for the main outcome. All three types of missingness were handled by excluding patients who did not have complete follow-up data. The assumption for this approach was that patients with complete outcome data were exchangeable with patients with missing data.

#### Strict censoring plus IPCW

In the second approach, we used strict censoring and time-varying inverse probability of censoring weighting (IPCW) [24]. Subjects were censored (set as “drop-out”) at the first visit with missing outcome data or at the first intermittent missing visit, whichever occurred first. This created a monotone missing pattern and allowed the use of IPCW to account for naturally occurring or created drop-out [24]. The IPCW method assigns weights to individuals with complete follow-up data corresponding to the inverse of their estimated probability of having complete data [24]. All approaches using IPCW assume a correctly specified IPCW model to account for missing data. We specified a logistic regression model to predict the probability of missing any of the variables required to calculate the outcome data. In the calculation of the IPCWs, we used both baseline values and time-varying values of relevant covariates at available visits to predict missingness. Further details on the IPCW-model, the selection of covariates included in the model and specification of the STATA code are available in supplementary file S5.

#### MI plus censoring plus IPCW

In the third approach, we used multiple imputation (MI) [25–27] in combination with IPCW. First, missing outcome data at completed visits were imputed using MI. Thereafter, subjects were censored at the first intermittent missing visit or the first missing visit due to drop-out, whichever occurred first. This created a monotone missing pattern and IPCW was used to account for missing outcome data due to naturally occurring or created drop-out. MI models missing outcome data given available information in the dataset. All approaches using MI assume a correctly specified MI model to account for missing data.

#### MI plus IPCW

In the fourth approach, we also used MI in combination with IPCW. First, we used MI to impute missing outcome data at completed visits and outcome data for intermittent missing visit. Thereafter, IPCW was applied to account for the remaining, naturally occurring drop-out in the imputed datasets.

#### MI for all missing

In a final approach, we used MI to impute all three types of missing outcome data: missing data at completed visits, intermittent missing visits, and drop-out. For all approaches involving MI, relevant available observations at all visits in each of the cohorts were used to inform the imputation models. Given the limited range and typically non-normal distribution of the variables to be imputed, we used multiple imputation by chained equations [27, 28]. We specified separate MI models and created 10 imputed datasets for each cohort, and these were combined into 10 final datasets. Estimates were averaged into a final estimate, while the standard errors were obtained using Rubin’s rule [25]. Further details on the MICE-models, the selection of covariates included in the models and specification of the STATA codes are available in supplementary file S6.

### Comparison metrics

We applied each of these five missing data approaches to conduct the comparative effectiveness study of treat-to-target strategies with different stringency. The main endpoint was binary and defined as achievement of remission or not according to the disease activity score in 28 joints (DAS28) (24). The DAS28 is a composite disease activity index with a score between 0 and 9.4. Remission is defined as a score < 2.6 (25). We first specified logistic regression models to compare the log odds ratio (OR) estimates, standard errors, and ORs for the treatment strategy outcomes across the five missing data approaches. STATA codes for the final outcome models are available in supplementary file S7. Finally, we compared the estimated proportions of patients in remission at 6, 12, 24 months according to each treatment strategy.

## Results

### Cohort characteristics

A total of 188 patients from the ARCTIC trial and 328 patients from the NOR-VEAC study met the common eligibility criteria (supplementary file S1).

### ARCTIC trial data

In the ARCTIC trial, 89.9% (169/188) of patients had complete follow-up data for the main outcome. Patients with incomplete follow-up data (drop-out exclusively) were younger, had less comorbidity, lower education, and more were current smokers compared to patients with complete follow-up data (supplementary file S8). Furthermore, patients with missing data also had higher disease activity at baseline, with a mean (standard deviation) DAS28 of 5.4 (1.5) compared with 4.7 (1.2) in patients with complete data, which gives a standardized mean difference of 0.448 (online supplementary file S8).

### NOR-VEAC observational study data

In the NOR-VEAC study, only 39.0% (128/328) of patients had complete follow-up data for the main outcome. Patients with incomplete follow-up data were somewhat younger and had lower education than patients with complete follow-up data; however, disease activity levels at baseline were similar (supplementary file S8).

### Description of longitudinal missing data patterns

**Fig. 1:**
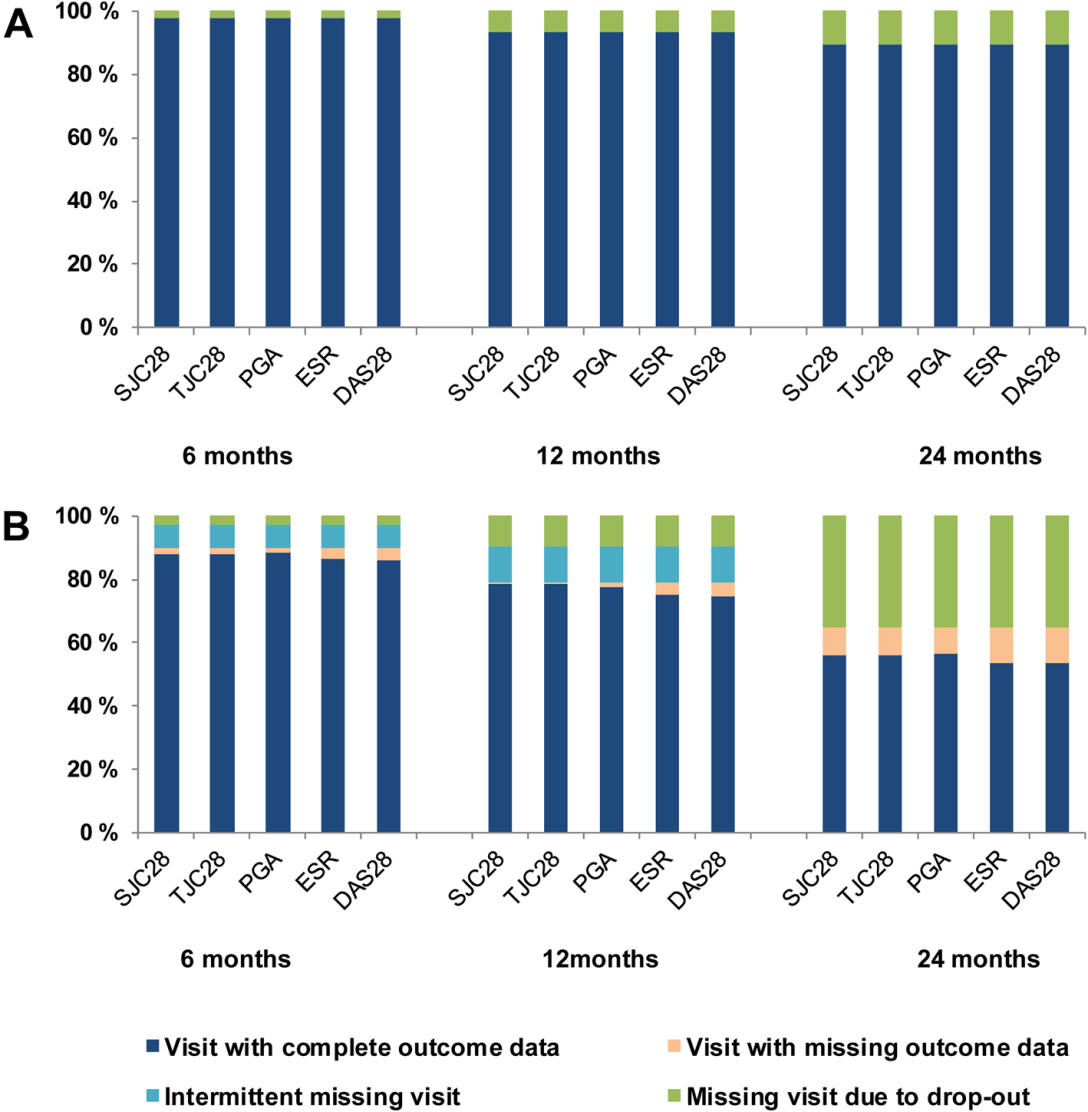
Patterns of missing outcome data in **A** the ARCTIC trial and **B** the NOR-VEAC observational study after standardization of follow-up. SJC28, swollen joint count in 28 joints; TJC28, tender joint count in 28 joints; PGA, patient’s global assessment of disease; ESR, erythrocyte sedimentation rate; DAS28, Disease Activity Score in 28 joints

Missing data in the ARCTIC trial was monotone and almost exclusively a result of drop-out. The drop-out rate was 1.6% at 6 months, 6.4% at 12 months, and 10.1% at 24 months (Fig. 1). In the NOR-VEAC observational study, the proportion of missing data was considerably higher, resulting from missing outcome data at completed visits, intermittent missing visits or drop-out, i.e., a non-monotone missing pattern (Fig. 1). Drop-out in NOR-VEAC counted for 2.4% of missing data at 6 months, 9.2% at 12 months and 35.1% at 24 months. Additionally, at the 6-month visit 7.3% of patients had an intermittent missing visit and 4.0% had missing outcome data at a recorded visit, while at the 12-month visit 11.3% had an intermittent missing visit, and 4.6% had missing outcome data at a recorded visit. At 24 months, 8.5% of patients had missing outcome data at a recorded visit (Fig. 1).

### Results from method comparison

#### Impact of missing data approaches on effect estimates

Regardless of the approaches to missing data (Table 2), the odds of achieving disease remission was higher for the “stringent treat-to-target” (based on the trial data) than for the “pragmatic treat-to-target” (based on the observational study data) in our target trial emulation.

**Table 2:** Differences in achievement of remission at 6, 12 and 24 months in the ARCTIC trial (Norway; 2010–2015) compared to the NOR-VEAC observational study (Norway; 2010–2018) according to different approaches to missing data^a^

Approaches to missing data	Estimate	SE	Odds ratio	95% CI	***p***-value
**6 months**					
CC^b^	0.756	0.288	2.13	1.21–3.75	0.009
Strict censoring + IPCW^c^	0.423	0.223	1.53	0.99–2.37	0.058
MI + censoring + IPCW^d^	0.401	0.222	1.49	0.97–2.31	0.072
MI + IPCW^e^	0.401	0.220	1.49	0.97–2.30	0.069
MI for all^f^	0.401	0.225	1.49	0.96–2.32	0.074
**12 months**					
CC^b^	1.093	0.294	2.98	1.68–5.31	< 0.001
Strict censoring + IPCW^c^	0.879	0.259	2.41	1.45–4.00	< 0.001
MI + censoring + IPCW^d^	0.797	0.250	2.22	1.36–3.62	0.001
MI + IPCW^e^	0.768	0.243	2.16	1.34–3.47	0.002
MI for all^f^	0.687	0.248	1.99	1.22–3.23	0.006
**24 months**					
CC^b^	0.429	0.288	1.54	0.87–2.70	0.136
Strict censoring + IPCW^c^	0.407	0.284	1.50	0.86–2.62	0.151
MI + censoring + IPCW^d^	0.448	0.279	1.57	0.91–2.70	0.108
MI + IPCW^e^	0.401	0.268	1.49	0.88–2.52	0.134
MI for all^f^	0.367	0.253	1.44	0.88–2.38	0.147

*SE* Standard error, *CI* Confidence interval, *CC* Complete case analyses, *IPCW* Inverse probability of censoring weighting, *MI* Multiple imputation

^a^For all approaches, inverse probability of treatment weighting using the propensity score was used to balance the two cohorts on relevant baseline covariates

^b^Analyses in a subset of patients with complete follow-up data for the main outcome

^c^Censoring of subjects with missing outcome data at a visit or intermittent missing visits, whichever occurred first. IPCW used to account for missing data due to drop-out (naturally occurring or created due to censoring)

^d^MI used to account for missing outcome data at visits. Censoring of subjects with intermittent missing visits. IPCW used to account for missing data due to drop-out (naturally occurring or created due to censoring)

^e^MI used to account for missing outcome data at a visit and intermittent missing visits. IPCW used to account for naturally occurring drop-out

^f^MI used to account for all missing data

The complete follow-up case analysis provided higher effect estimates than the more sophisticated approaches to missing data. This tendency was most apparent at the 6-month assessment, when the OR estimate was 2.13 [95% confidence interval (CI) 1.21, 3.75] favoring the stringent treat-to-target strategy. Other approaches to missing data gave more conservative estimates ranging from OR 1.53 [95% CI 0.99, 2.37] for the strict censoring plus IPCW approach to OR 1.49 [95% CI 0.96, 2.32] for the MI for all missing approach. All three approaches involving MI to various extents yielded essentially identical results for the 6-month assessment. Statistical efficiency was evidently worse (higher standard errors) for the complete follow-up case analysis, which handled all missingness by exclusion of patients.

The 12-month assessment generally gave similar results, with the complete follow-up case approach giving the most optimistic results, whereas the other more sophisticated approaches giving more conservative and similar results. The estimates became slightly more conservative as the extent of MI use increased from the strict censoring plus IPCW approach (no MI; OR 2.41 [95% CI 1.40, 4.00]) to the MI for all missing approach (OR 1.99 [95% CI 1.22, 3.23]).

The 24-month assessment generally exhibited a similar trend to the 6- and 12-month assessments, but the discrepancies between all approaches were subtle (OR 1.44–1.57) compared with the first two time points. Since there were no intermittent missing visits at 24 months, the two combinations of MI and IPCW both used MI to impute partial missing visit data and IPCW to account for naturally occurring drop-out.

#### Impact of missing data approaches on response rate

**Fig. 2:**
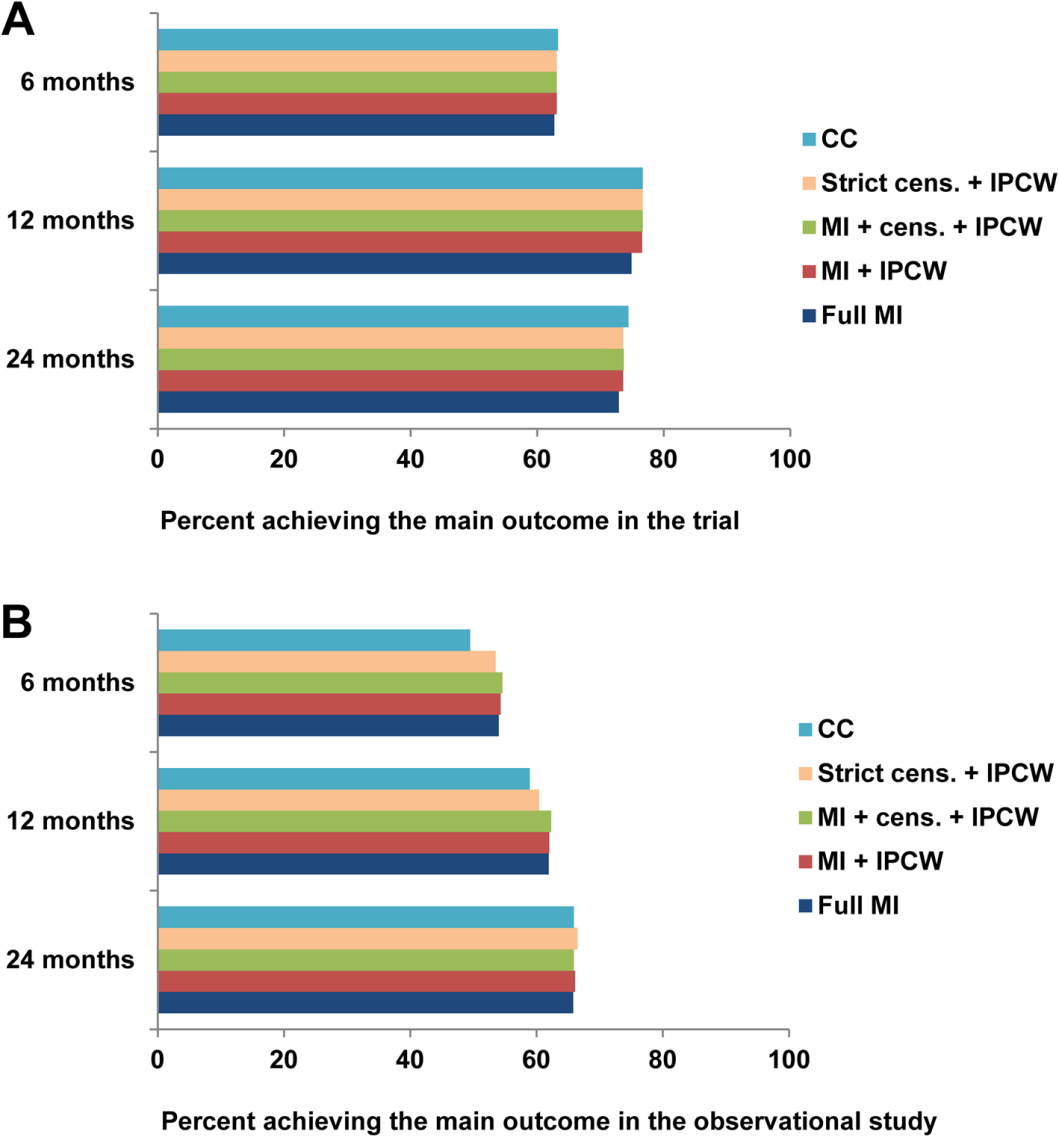
Estimated proportion of patients achieving the main outcome according to different approaches to missing data in **A** the ARCTIC trial and **B** the NOR-VEAC observational study CC, complete (follow-up) case; cens., censoring; IPCW, inverse probability of censoring weighting; MI, multiple imputation

The estimated proportion achieving disease remission in the emulated “stringent treat-to-target” arm (based on the trial data) was similar across the approaches to missing data (Fig. 2). Since missing data in the trial were almost exclusively due to drop-out, the estimated proportion achieving remission was not affected by the different approaches to partial missing visit data or intermittent missing visit. This implies that all approaches including IPCW used this method to account for naturally occurring drop-out only. The estimated proportions achieving remission were somewhat lower at 12 and 24 months when using MI to account for drop-out (Fig. 2). Compared to the remission rate among observed data points, the imputed data points for drop-out in the trial gave a somewhat lower mean remission rate at 12 months (supplementary file S9), which is consistent with higher baseline disease activity in patients with incomplete follow-up data (supplementary file S8).

In the emulated “pragmatic treat-to-target” arm (based on the observational study data), the estimated proportions achieving disease remission varied across different approaches to missing data at 6 and 12 months, but were similar at 24 months (Fig. 2). At 6 and 12 months, analyses of patients with complete follow-up data and the approach using strict censoring and IPCW resulted in lower estimated proportions achieving the main outcome than the approaches handling incomplete visit data and intermittent missing visits in addition to drop-out (Fig. 2). Since there were no intermittent missing visits at 24 months, the two combinations of MI and IPCW both used MI to impute partial missing visit data and IPCW to account for naturally occurring drop-out. In the observational data, imputed data points gave similar mean disease remission rates compared to the remission rates among observed data points, regardless of the extent of imputations (supplementary file S9).

## Discussion

We examined the impact of five alternative approaches to longitudinal missing data in an empirical example of RA treatment strategy comparison, in which we used data from a prospective observational study to form an external control group for a clinical trial. We consistently found superior outcomes of the “stringent treat-to-target” strategy (based on the trial data) compared with the “pragmatic treat-to-target” strategy (based on the observational study data), although the difference was only statistically significant at the 12-month visit. The complete follow-up case analysis tended to give higher effect estimates of the OR and wider confidence intervals compared with the other more sophisticated approaches; however, this difference decreased with longer follow-up. The four methods employing IPCW, MI, and their combinations, generally yielded similar OR estimates despite their differing modeling assumptions.

Although the literature on missing data is vast [26], our paper is unique in its focus on longitudinal missing data challenges in the emerging area of using real-world data as external controls for trial data [4]. The similarity of the patient populations and applied treatment strategies in the two studies providing data for this methodological investigation enabled us to assess missing data patterns resulting from follow-up under different study designs. We found a larger amount of missing data with more complex missing patterns in the observational study compared with the limited and monotone missing data in the trial. As a result, the differences across the alternative approaches to longitudinal missing data mainly came from the estimated proportions of disease remission in the “pragmatic treat-to-target” strategy arm (based on the observational study data). Most notably, the complete follow-up case analyses gave smaller estimates for the proportions of patients reaching the desired remission outcome at 6 and 12 months in the “pragmatic treat-to-target” strategy arm. This made the estimated benefits associated with the “stringent treat-to-target” strategy (based on the trial data) appear better. The difference among the advanced missing data approaches mainly came from the extent of IPCW use rather than changes in imputed values when MI was used more extensively.

Both IPCW and MI can provide unbiased estimates under the missing at random assumption, which is weaker than the assumption for the complete follow-up case analysis. An advantage of MI is that this method efficiently uses information from individuals with partially missing data [24, 25, 27]. All available and relevant data can be included in the imputation model, including both variables related to the outcome analyses and variables associated with missingness [25, 27]. However, the MI approach is potentially sensitive to misspecification in situations where some individuals have large blocks of missing values [24]. Thus, missing data due to drop-out in the present study may make MI less appealing, especially for the 24- month time point. IPCW assumes a correctly specified model for the missingness mechanism, given observed data at previous time points [24, 29]. A correctly specified IPCW can account for missingness due to blocks of drop-out. However, IPCW can be less efficient due to the loss of information from incomplete cases [24]. Thus, the IPCW model for the trial data, with smaller amounts of missing at 6 and 12 months and a maximum of 10.1% missing at 24 months, was likely to be more efficient than the IPCW model for the observational data, with a substantial amount of missing outcome data during follow-up.

In the present empirical evaluation, censoring all patients at first missing data (strict censoring) created a monotone missing pattern in the observational data, while a monotone missing pattern already existed naturally in the trial data. Despite using IPCW to account for created or naturally occurring drop-out, the estimates from the strict censoring approach were less efficient at 12 and 24 months than approaches involving MI, reflecting the substantial loss of information due to excluded data points. This may indicate increased efficiency due to recovered information when using MI to impute all or partial missing visit data and may be preferable compared with excluding individuals at first missing value.

A limitation of this methodological investigation is the generalizability of results to other settings using an external control group. Data in the external control group of the present study was provided by a contemporaneous, prospective observational study with a patient population initiating treatment and follow-up strategy similar to the trial [12–14]. This is the most favorable type of external control group [4]. As a result, emulating a target trial was relatively straightforward. In a more typical use of synthetic control arms, the comparator arm may not receive new medication, device, or treatment, introducing additional challenges, such as ambiguity of time zero (start of follow-up). Sources of observational data, such as electronic health records and insurance claims, likely pose more missing data challenges than seen in our observational data source due to the complete lack of recommended follow-up frequencies. Finally, as we used empirical data rather than simulations, we do not know the true underlying effect of the “stringent treat-to-target” compared to “pragmatic treat-to-target”. Our empirical evaluation was limited to methods assuming missing-at-random. Additional consideration of missing-not-at-random may be reasonable in practice. Under this general missingness mechanism, observed data alone is not enough for unbiased missingness handling, requiring a range of sensitivity analyses. MI may be more approachable for this purpose as some MI software allows shifting of imputed values by a user-specified sensitivity parameter (this “delta adjustment” specifies the departure from MAR) during the imputation process [26, 30]. With the IPW approach, sensitivity analyses focused on the missing probability model could be performed, although the interpretability is not as transparent [31].

## Conclusions

In conclusion, we empirically examined the impact of different approaches for missing follow-up data when using data from an observational study to form an external control arm for a clinical trial. Despite the favorable setting of having prospectively collected observational data, there were some differences in the effect estimates although the clinical conclusion was not affected qualitatively. The differences mainly came from the handling of more extensive and complex missing data in the observational data source. When using routine observational data as external controls even more complex missingness issues are likely expected. As the quality of a comparative effectiveness study is dependent on what we compare to, we cannot overemphasize the importance of carefully examining missing data patterns across data sources, using a prespecified advanced missing data method beyond simply using complete follow-up. In the current study, using MI for all missing outcome data at visits, intermittent missing visits, and drop-out, was the most pragmatic approach, providing the simplicity of using a single missing data method and a potential efficiency gain due to the ability of using information from individuals with partial missing data. However, conducting sensitivity analyses with alternative missing data methods is recommended. Particularly in settings with high drop-out rates, IPCW may be a valuable addition.

## Supplementary Information

**Additional file 1.**

## Data Availability

The datasets used for the current methodological investigation contain sensitive patient information and restrictions apply to the availability of the data. For this reason, these datasets are not publicly available. However, relevant anonymized data can be made available on reasonable request to the corresponding author, after appropriate approvals.

## Abbreviations

CI: Confidence interval
DAS28: The disease activity score in 28 joints
DMARD: Disease-modifying anti-rheumatic drug
IPCW: Inverse probability of censoring weighting
MI: Multiple imputation
OR: Odds ratio
RA: Rheumatoid arthritis
RCT: Randomized controlled trial
