# Supplementary material for: Using observational study data as an external control group for a clinical trial: an empirical comparison of methods to account for longitudinal missing data
Source: BMC Med Res Methodol. 2022 May 28;22:152. doi: 10.1186/s12874-022-01639-0 (PMC9148529; doi:10.1186/s12874-022-01639-0)
Supplement: Supplementary file 1 — Additional file 1. [file 12874_2022_1639_MOESM1_ESM.docx]

**Supplementary file S1**. Overview of inclusion and exclusion of patients from the ARCTIC trial and the NOR-VEAC observational study according to the eligibility criteria and treatment strategy specified in the hypothetical target trial. RA, rheumatoid arthritis; MTX, methotrexate; DMARD, disease modifying anti-rheumatic drug.

**

**

| **Supplementary file S2** Specification and emulation of a target trial designed to compare stringent and pragmatic treat-to-target strategies for rheumatoid arthritis (RA). Target trial emulation with data from the ARCTIC trial (Norway; 2010-2015) and the NOR-VEAC observational study (Norway; 2010-2018). | | | |
| --- | --- | --- | --- |
| **Component** | **Target trial** |  | **Target trial emulation** |
| Eligibility criteria | Adult treatment-naïve patients fulfilling the 2010 classification criteria for rheumatoid arthritis and with less than 13 months since first manifestation of disease. |  | Same |
| Treatment strategies | Arm 1) Stringent treat-to-target strategy  Arm 2) Pragmatic treat-to-target strategy  Patients in both arms start with methotrexate monotherapy as their first disease modifying drug therapy |  | Arm 1) Patients from the ARCTIC trial who met the common eligibility criteria  Arm 2) Patients from the NOR-VEAC observational study who met the common eligibility criteria.  Patients in both arms started with methotrexate monotherapy as their first disease modifying drug therapy |
| Treatment assignment | Patients are randomly assigned to either strategy |  | Individuals were classified to stringent or pragmatic treat-to-target strategies according to the data sources: the ARCTIC trial (stringent) or the NOR-VEAC observational study (pragmatic).  Baseline randomization was emulated by using inverse probability of treatment weighting of baseline covariates. |
| Follow-up plan | Follow-up starts at initiation of methotrexate and ends at loss to follow-up or two years after baseline, whichever occurs first.  Assessments and calculation of outcome at visits at 6, 12 and 24 months after baseline. |  | Follow-up started at initiation of methotrexate and ended at loss to follow-up or two years after baseline, whichever occurred first.  Follow-up schedules in ARCTIC and NOR-VEAC were standardized to include follow-up visits at 6, 12 and 24 months. |
| Outcome | Remission defined by the Disease Activity Score in 28 joints (composite score of tender joint count [0–28], swollen joint count [0–28], patient's global assessment [0–10], and inflammatory marker) at the time points in the follow-up plan. |  | Same |
| Causal contrast | Intention-to-treat effect, i.e. the effect of being assigned to either the stringent treat-to-target strategy or to the pragmatic treat-to-target strategy. |  | Observational analogue of intention-to-treat effect, i.e. the effect of initiating treatment in either the ARCTIC trial or in the NOR-VEAC study. |
| Statistical analyses | Modified intention-to-treat analysis, i.e. accounting for loss to follow-up using different approaches |  | Observational analogue of modified intention-to-treat analyses, using different approaches to account for missing data |

**Supplementary File S3.** Inverse probability of treatment weighting (IPTW).

We used IPTW using the propensity scores calculated from baseline covariates to emulate randomization to either the ARCTIC trial or the NOR-VEAC observational study. The IPTW model and selection of covariates are explained and specified in the main text. The following section provide some additional details on the balancing of baseline covariates.

We used the average treatment effect on the treated weighting scheme, handling the ARCTIC group as “treated”, with the aim of estimating the strategy effect in a group of RA patients having a covariate distribution as in the ARCTIC trial [1]. Standardized mean differences were calculated for all covariates before and after applying propensity score weighting to assess the balance of baseline covariates between the two cohorts. Differences less than 0.1 were considered negligible [2].

The following code was applied using the statistical software STATA, version 16.0:

logit *cohort_affiliation* *var1 var2 ………… var16*

predict pscore,pr

generate ipw=1/pscore if *cohort_affiliation* =1

replace ipw=1/(1-pscore) if *cohort_affiliation* =0

logit study

predict num, pr

gen sw=num/pscore if *cohort_affiliation* =0

replace sw=(1-num)/(1-pscore) if *cohort_affiliation* =0

1. Sato T, Matsuyama Y. Marginal structural models as a tool for standardization. Epidemiology. 2003;14(6):680-686.
2. Austin PC, Stuart EA. Moving towards best practice when using inverse probability of treatment weighting (IPTW) using the propensity score to estimate causal treatment effects in observational studies. Stat Med. 2015;34:3661-79.

**Supplementary File S4.** Examples of three types of missingness.





**Supplementary File S5.** Inverse probability of censoring weighting (IPCW).

IPCW was used to account for monotone missing outcome data during follow-up. While the missing pattern in the ARCTIC trial was naturally monotone, the missing pattern in the NOR-VEAC observational study was non-monotone. Therefore, prior to the calculation of IPCWs, a monotone missing pattern was created in the NOR-VEAC dataset by applying strict censoring and/or multiple imputation of missing outcome data at visits and intermittent missing visits.

To calculate the IPCWs we specified a logistic regression model with regard to the probability of data being missing. We included the following baseline covariates considered to to be related to missingness: cohort affiliation (the ARCTIC trial versus the NOR-VEAC observational study), age, gender, months since first swollen joint, higher education (completed college or university degree), rheumatoid factor positivity, anti-cyclic citrullinated peptide positivity, number of comorbidities (≥1 versus none), smoking status (never/previous versus current), erythrocyte sedimentation rate (ESR), patient´s global assessment of disease (PGA), swollen joint count in 28 joints (SJC28) and tender joint count in 28 joints (TJC28). For calculation of the denominator of the censoring weights (*censdenom*), we used both baseline values and time-varying values of the following covariates: ESR, PGA, SJC28 and TJC28. The numerator of the censoring weights (*censnum*) was calculated as a stabilizing factor to reduce the variance of the final weights and was fitted with a similar logistic regression containing only baseline values. The final weights were defined as *censnum/censdenom*.

The following code was applied using the statistical software STATA, version 16.0:

logistic *censvar* *cohort_affiliation baselinevar1 ….. baselinevar13 esr esr_lag1 pga pga_lag1 sjc28 sjc28_lag1 tjc28 tjc28_lag1 time*

predict pcens if e(sample)

replace pcens=1-pcens

bys patient_id: replace pcens=pcens*pcens[_n-1] if _n!=1

rename pcens censdenom

logistic *censvar* *cohort_affiliation baselinevar1 ….. baselinevar13*

predict pcens if e(sample)

replace pcens=1-pcens

bys patient_id: replace pcens=pcens*pcens[_n-1] if _n!=1

rename pcens censnum

gen censweight=censnum/censdenom

**Supplementary File S6.** Multiple imputation by chained equations (MICE).

Missing data at visits and intermittent missing visits were only present in the NOR-VEAC observational study. We specified a common imputation model to impute missing outcome data at visits, missing outcome data at intermittent missing visits and missing outcome data due to drop-out, using the full NOR-VEAC dataset (n=328) with assessment at baseline, 3, 6, 12, 16, 20 and 24 months to inform the imputation model. Covariates were included into the inputation model if considered to be releted to the main analyses or to missingness. The following covariates used to calculate the outcome of DAS28 remission were imputed: erythrocyte sedimentation rate (ESR), swollen and tender joint count in 28 joints (SJC28 and TJC28) and patient global assessment (PGA). Furthermore, the following covariates with incomplete follow-up assessments, but considered relevant to inform the imputation of missing outcome data, were included in the model as covariates to be imputed: C-reactive protein (CRP), physician global assessment (PHGA), fatigue (VAS 0-100) and the EQ-5D-index score*.* Additionally, the following auxiliary variables were included in the model: age, gender, months since first swollen joint, higher education (completed college or university degree), rheumatoid factor positivity, anti-cyclic citrullinated peptide positivity, number of comorbidities (≥1 versus none) and smoking status (never/previous versus current).

The imputations of variables were performed using the statistical software STATA, version 16.0. Data were clustered on patient-ID and ten imputed datasets were created.

reshape wide *var1 var2 ……var16*, i(patient_ID) j(time)

mi set wide

mi register imputed *var1 var2 ….var8*

mi register regular *var9 var10 ….var16*

mi impute chained (pmm, knn(5)) *var1-var8* = *var9 var10 ….var16* , add(10) rseed(*n*) burnin(20) savetrace(extrace, replace)

mi reshape long *date visit var1 var2 …. var8*, i(patient_ID) j(time)

Each of the ten imputed datasets were duplicated and stored in three different versions: one containing only the imputed missing outcome data at visits, one containing imputed missing outcome data at visits plus imputed outcome data at intermittent missing visits, and one where all missing outcome data had been imputed. The two former versions were combined with the original ARCTIC dataset for analyses.

We specified an identical model to impute missing outcome data due to drop-out in the ARCTIC study, using the full ARCTIC dataset (n=188) and including assessment at baseline, 3, 6, 12, 16, 20 and 24 months to inform the imputation model. Each of the ten imputed ARCTIC datasets were combined with one of the NOR-VEAC datasets where all missing outcome data had been imputed (ARCTIC imputed dataset 1 to NOR-VEAC imputed dataset 1; ARCTIC imputed dataset 2 to NOR-VEAC imputed dataset 2, etc.).

After running the outcome analyses in ten combined datasets for each level of imputed outcome data (missing data at visit, missing data at visit plus intermittent missing visits, all missing data), the obtained estimates were averaged into a final estimate, while the standard errors were obtained using Rubin´s rules [1].

1. Rubin DB. Multiple Imputation for Nonresponse in Surveys. New York, NY: John Wiley & Sons, Inc., 1987.

**Supplementary File S7**. STATA codes for final outcome models.

We modeled the probability of achieving remission at 6, 12 and 24 months. Baseline balancing using inverse probability of treatment weighting was applied for all missing data approaches, including the complete case analyses. For the approaches including inverse probability of censoring weighting (IPCW) to account for missing data, the two weights were combined into one.

In the complete case analyses, the outcome model included the following independent covariates in addition to cohort affiliation: age, gender, months since first swollen joint, higher education (completed college or university degree), number of comorbidities (≥1 versus none) and the baseline values of erythrocyte sedimentation rate (ESR), patient´s global assessment of disease (PGA), swollen joint count in 28 joints (SJC28) and tender joint count in 28 joints (TJC28).

For the remaining four approaches, the outcome models included the following independent covariates in addition to cohort affiliation: age, gender, months since first swollen joint and the baseline values of ESR, PGA, SJC28 and TJC28.

The following codes were applied using the statistical software STATA, version 16.0.

**Complete case analyses**

logit *remission cohort_affiliation* *independentvar2…..independentvar10* [pw=sw]

**Three approaches involving IPCW**:

Strict censoring + IPCW; multiple imputation (MI) + censoring + IPCW; MI + IPCW

logit *remission cohort_affiliation* *independentvar2…..independentvar8* [pw=sum_weight], cluster(patient-ID)

**One approach using MI to account for all missing outcome data**

logit *remission cohort_affiliation* *independentvar2…..independentvar8* bl [pw=sw], robust

| **Supplementary file S8** Differences in baseline characteristics in patients with complete versus missing follow-up data in the ARCTIC trial and in the NOR-VEAC observational study | | | | | | |
| --- | --- | --- | --- | --- | --- | --- |
|  | **ARCTIC** | | | **NOR-VEAC** | | |
|  | **Complete data** | **Missing data** | **Std.diff** | **Complete data** | **Missing data** | **Std.diff** |
| **Baseline characteristics^1^** | **n=169** | **n=19** |  | **n=128** | **n=200** |  |
| Age, years | 52.0 (13.4) | 43.4 (16.5) | 0.570 | 55.4 (12.5) | 52.8 (14.3) | 0.193 |
| Female | 61.5 | 57.9 | 0.074 | 68.0 | 65.0 | 0.063 |
| Anti-CCP positive | 81.7 | 84.2 | 0.068 | 78.1 | 74.0 | 0.097 |
| Rheumatoid factor positive | 68.6 | 94.7 | 0.717 | 64.1 | 63.0 | 0.022 |
| Months from first swollen joint | 5.5 (4.1) | 5.8 (3.2) | -0.090 | 4.5 (2.9) | 4.5 (2.7) | 0.008 |
| Presence of ≥1 comorbidity | 58.6 | 52.6 | 0.120 | 40.6 | 43.5 | 0.058 |
| Current smoker | 20.7 | 31.6 | 0.249 | 21.1 | 25.0 | 0.093 |
| University or college degree | 42.0 | 36.8 | 0.106 | 53.1 | 45.0 | 0.163 |
| CRP, mg/L | 15.9 (22.3) | 19.9 (22.8) | -0.175 | 20.2 (24.4) | 19.3 (27.9) | 0.035 |
| ESR, mmHg | 25.0 (19.4) | 30.0 (19.7) | -0.258 | 28.6 (21.1) | 27.1 (20.0) | 0.071 |
| Swollen joint count in 28 joints | 7.0 (5.5) | 9.2 (6.9) | -0.342 | 6.3 (4.9) | 6.2 (5.4) | 0.025 |
| Tender joint count in 28 joints | 6.9 (4.8) | 9.2 (5.9) | -0.434 | 6.5 (5.2) | 6.6 (5.9) | -0.025 |
| Physician´s global assessment | 40.1 (20.1) | 54.1 (21.5) | -0.670 | 40.5 (18.9) | 41.1 (20.0) | -0.030 |
| Patient´s global assessment | 49.5 (24.3) | 59.0 (18.1) | -0.445 | 47.7 (25.2) | 50.6 (24.7) | -0.116 |
| DAS28 | 4.7 (1.2) | 5.4 (1.5) | -0.448 | 4.8 (1.3) | 4.7 (1.3) | 0.052 |
| EQ-5D | 0.522 (0.290) | 0.397 (0.310) | 0.419 | 0.501 (0.307) | 0.481 (310) | 0.065 |
| ^1^Mean (standard deviation) for continuous variables and percent for categorical variables.  Std.diff., standardized mean difference; anti-CCP, anti-cyclic citrullinated peptide positivity, CRP, C-reactive protein; ESR, erythrocyte sedimentation rate; DAS28, Disease Activity Score in 28 joints; EQ-5D, EuroQol-5 Dimensions | | | | | | |

| **Supplementary File S9.** Achievement of DAS28^1^ remission among observed and imputed subjects^2^ in the NOR-VEAC observational study and in the ARCTIC trial | | | | | | | |
| --- | --- | --- | --- | --- | --- | --- | --- |
|  | **NOR-VEAC** | | | |  | **ARCTIC** | |
|  |  | **Imputed variables at visits**  (approach: MI+censoring+IPCW) | **Imputed variables at visit + intermittent missing visits**  (approach: MI+IPCW) | **Imputed variables at visit + intermittent missing visits +**  **drop-out**  (approach: MI for all missing) |  |  | **Imputed drop-out**  (approach: MI for all missing) |
|  | **Observed** | **Imputed** | **Imputed** | **Imputed** |  | **Observed** | **Imputed** |
| **6 months** |  |  |  |  |  |  |  |
| N (%) | 283 | 13 (4.4) | 40 (12.4) | 45 (13.7) |  | 185 | 3 (1.6) |
| DAS28^1^ remission, (%) | 53.0 | 57.6 | 54.3 | 54.7 |  | 63.8 | 60.0 |
| **12 months** |  |  |  |  |  |  |  |
| N (%) | 246 | 15 (5.8) | 66 (21.2) | 82 (25.0) |  | 176 | 12 (6.4) |
| DAS28^1^ remission, % | 61.4 | 63.3 | 63.2 | 62.7 |  | 80.7 | 75.8 |
| **24 months** |  |  |  |  |  |  |  |
| N (%) | 176 | 37 (17.4) | - | 152 (46.3) |  | 169 | 19 (10.1) |
| DAS28^1^ remission, % | 65.3 | 65.1 | - | 65.6 |  | 78.1 | 80.5 |
| DAS28, Disease Activity Score in 28 joints  ^2^Remission rates were averaged across imputed datasets | | | | | | | |
